# Innovative Diagnostic Endoscopy in Inflammatory Bowel Diseases: From High-Definition to Molecular Endoscopy

**DOI:** 10.3389/fmed.2021.655404

**Published:** 2021-07-21

**Authors:** Christian Bojarski, Maximilian Waldner, Timo Rath, Sebastian Schürmann, Markus F. Neurath, Raja Atreya, Britta Siegmund

**Affiliations:** ^1^Charité–Universitätsmedizin Berlin, Corporate Member of Freie Universität Berlin, Humboldt-Universität zu Berlin, and Berlin Institute of Health, Department for Medicine (Gastroenterology, Infectious diseases, Rheumatology), Berlin, Germany; ^2^Department of Medicine 1, Friedrich-Alexander-Universität Erlangen-Nürnberg, Erlangen, Germany; ^3^Department of Chemical and Biological Engineering, Institute of Medical Biotechnology, Friedrich-Alexander-University Erlangen-Nürnberg, Erlangen, Germany; ^4^Deutsches Zentrum Immuntherapie DZI, Erlangen, Germany

**Keywords:** high-definition endoscopy, confocal laser microscopy, chromoendoscopy, molecular endoscopy, multiphoton microscopy

## Abstract

High-definition endoscopy is one essential step in the initial diagnosis of inflammatory bowel disease (IBD) characterizing the extent and severity of inflammation, as well as discriminating ulcerative colitis (UC) from Crohn's disease (CD). Following general recommendations and national guidelines, individual risk stratification should define the appropriate surveillance strategy, biopsy protocol and frequency of endoscopies. Beside high-definition videoendoscopy the application of dyes applied via a spraying catheter is of additional diagnostic value with a higher detection rate of intraepithelial neoplasia (IEN). Virtual chromoendoscopy techniques (NBI, FICE, I-scan, BLI) should not be recommended as a single surveillance strategy in IBD, although newer data suggest a higher comparability to dye-based chromoendoscopy than previously assumed. First results of oral methylene blue formulation are promising for improving the acceptance rate of classical chromoendoscopy. Confocal laser endomicroscopy (CLE) is still an experimental but highly innovative endoscopic procedure with the potential to contribute to the detection of dysplastic lesions. Molecular endoscopy in IBD has taken application of CLE to a higher level and allows topical application of labeled probes, mainly antibodies, against specific target structures expressed in the tissue to predict response or failure to biological therapies. First pre-clinical and *in vivo* data from label-free multiphoton microscopy (MPM) are now available to characterize mucosal and submucosal inflammation on endoscopy in more detail. These new techniques now have opened the door to individualized and highly specific molecular imaging in IBD in the future and pave the path to personalized medicine approaches. The quality of evidence was stated according to the Oxford Center of evidence-based medicine (March 2009). For this review a Medline search up to January 2021 was performed using the words “inflammatory bowel disease,” “ulcerative colitis,” “crohn's disease,” “chromoendoscopy,” “high-definition endoscopy,” “confocal laser endomicroscopy,” “confocal laser microscopy,” “molecular imaging,” “multiphoton microscopy.”

## Introduction

Gastrointestinal endoscopy plays a crucial role in patients with inflammatory bowel disease (IBD; Crohn's disease CD; ulcerative colitis UC). The initial diagnosis, the determination of disease activity and surveillance are the key steps of rational disease management and include primarily an endoscopic approach to visualize and characterize the extent and severity of mucosal inflammation and to take targeted biopsies of inflamed and non-inflamed tissue areas. High-resolution or high-definition endoscopy should be the gold standard when examining IBD patients. In surveillance colonoscopy, a combination of high-definition endoscopy with classical dye-based chromoendoscopy (e.g., indigocarmine solution 0.1–0.5%) is of additional value to detect flat polypoid neoplastic mucosal lesions and discriminate these areas from colitis-associated pseudopolyps or other benign lesions (level 1, grade of recommendation A). The exclusion or detection of intraepithelial neoplasia (IEN) is the aim of all surveillance colonoscopies in IBD to reduce the risk of malignant transformation to colorectal cancer. Although many study data showed different results this risk seems to be lower than previously assumed (1) and is in the range of 1–7% after 10 and 30 years of UC, respectively (2), (level 2, grade B). In patients with CD the risk of developing colorectal cancer is lower than in UC, but still heightened with an incidence rate of 2% after 30 years (2), (level 2, grade B). The detection rate of IEN can be further improved by using *in-vivo* histology techniques. Confocal laser endomicroscopy (CLE) was introduced in 2006 and gave exclusive insight into the gastrointestinal tract on a cellular and subcellular level in a variety of gastrointestinal diseases. Initially, there were two independent *in-vivo* histology systems available on the market, the endoscope-based CLE (eCLE) by Pentax, Tokyo, Japan, and a probe-based CLE (pCLE) by Mauna Kea Technologies, Paris, France. A couple of years ago the technical support for eCLE was permanently discontinued and research activities with that specific system were restricted to a very small number of research centers with active running systems. In IBD, CLE was used for characterization and classification of inflammatory activity and mucosal healing in active disease as well as for the detection of IEN during surveillance. For example, a combination of chromoendoscopy with CLE can detect 5-fold higher rates of IEN compared with random biopsy protocols (3), (level 4 grade C). After evaluation of CLE as a unique tool for the characterization of normal, inflamed and pre-malignant or malignant intestinal mucosa, some research groups focused on the analysis of the intestinal barrier function for predicting clinical relapse (4), (level 3, grade C). Mucosal healing can predict response to therapy or, vice versa, ongoing mucosal or submucosal inflammation may indicate treatment failure. Kiesslich et al. published a study investigating epithelial barrier function by CLE and described leakage of fluorescein due to epithelial gaps during cell shedding (5), (level 3, grade C). Based on these and other data, highly specific fluorescein-labeled probes binding to their molecular targets on the surface of the gastrointestinal epithelium established a fascinating new era of molecular imaging studies. Molecular endoscopy allows a more specific and individual treatment by predicting the response to anti-inflammatory therapy (6), (level 3, grade C). Recently label-free multiphoton microscopy based on endogenous autofluorescence visualized mucosal inflammation in human biopsies of CD patients (7, 8).

## High-Definition Endoscopy and Chromoendoscopy in IBD

Lower optical resolution of previous endoscope generations and random biopsy protocols in all patients were central elements in the surveillance of IBD during the first decade of this century. The lower image quality might be one reason for the increased rate of colorectal cancers described earlier in UC patients (1). High-definition endoscopes have an average diameter of 9–13 mm, a field of view between 140 and 170°, an optical resolution up to 2 million pixels and a 4-way angulation and the newest generation of endoscopes is mostly equipped with bright LEDs (9). Over the last 10 years a more specific and, moreover, individual endoscopic strategy was implemented in national IBD guidelines focusing on defined risk factors. In Germany, surveillance colonoscopy in UC starts 6–8 years after initial diagnosis and should be performed between each year in high-risk patients and every 4 year in patients with low-risk conditions (10), (level 1, grade A). Recently patients with primary sclerosing cholangitis (PSC), a tubular colon and those with a history of neoplasia were identified as having a higher risk for developing colorectal cancer and in these patients targeted and additional random biopsies were recommended during chromoendoscopy (11), (level 1, grade A). For classical chromoendoscopy in the colon, either indigo carmine as a contrast enhanced dye or methylene blue as an absorptive dye can be used, for both agents a 0.1–0.5% working solution is recommended and should be applied with slight pressure via a spraying catheter to the mucosal surface to ensure optimal distribution throughout the entire colon. An adequate withdrawal time and sufficient bowel preparation (Boston Preparation Scale ≥6) is mandatory for an optimal view of the complete colonic mucosal surface. Huge efforts were made to investigate if virtual chromoendoscopy techniques (NBI, FICE, I-scan) are able to replace classical dye-based chromoendoscopy. NBI can characterize histological inflammation by the determination of mucosal vascular pattern (12), (level 4, grade C). This was recently confirmed and prediction of mucosal proliferation can be helpful in the diagnosis of IEN (13), (level 4, grade C). However, the inconsistent results of various studies currently do not justify the application of virtual chromoendoscopy as a single surveillance strategy (14, 15), (level 3, grade D). Studies favoring virtual chromoendoscopy found that the examination time and the technical efforts were significantly lower and therefore more user-friendly compared to the application of classical dyes via spraying catheter (16), (level 1, grade A). A new meta-analysis identified 11 randomized-controlled trials with a total of 1328 patients and concluded that virtual chromoendoscopy is as good as high-definition endoscopy with dye-based chromoendoscopy (17). This indicates that probably in a couple of years both techniques can be applied equally depending on the local expertise of the respective endoscopy unit. As we already know from our daily practice, the acceptance rate of classical chromoendoscopy among physicians is low. Therefore, a promising future perspective for any screening colonoscopy might be the pre-interventional intake of oral chromoendoscopy dye. Recently, the results of a phase 3 trial found an increase in the adenoma detection rate of 8.5% when peroral methylene blue tablets (MMX®) were administered together with bowel preparation (18), (level 1, grade A). Further studies will evaluate oral chromoendoscopy in patients with the need for recurring endoscopic surveillance colonoscopies.

## Confocal Laser Endomicroscopy (eCLE, pCLE)

Today we can look back on 15 years of confocal laser endomicroscopy (CLE). This exciting technique was originally designed to allow virtual histology on a cellular and subcellular level with the potential to at least partially replace classical histology. The procedure, however, is time-consuming, technically challenging and intravenous applied fluorescein is necessary for each procedure to generate high-resolution images. This and the fact that no reimbursement was provided by health care authorities restricted the running CLE systems to large research units in University centers. The acquisition of targeted biopsies became reality and a large number of clinical studies investigating a variety of gastrointestinal diseases were published between 2005 and 2012. Most of these studies characterized pre-malignant or inflammatory lesions in Barrett's esophagus (19), gastric cancer (20), celiac disease (21), IBD (22), graft-vs. host disease (23) or adenomatous polyps (24) in the upper and lower gastrointestinal tract (level 3, grade C). A fascinating overview of different cellular and subcellular pathologies was provided and after an initial characterization period the next level of CLE research was reached by explaining functional dynamic changes within the intestinal mucosa. The identification of epithelial gaps during cell shedding and the increase in gaps after stimulation with tumor necrosis factor (TNF) alpha caused loss of barrier function and integrity (5), (level 3, grade C). In IBD patients in clinical remission, increased cell shedding with fluorescein leakage was observed and associated with subsequent relapse 12 months after initial CLE (22) indicating that CLE is able to relapse or can define a stable disease when the barrier function is intact (level C, grade C). These observations were in accordance with electrophysiological measurements in human biopsies of patients with CD as described earlier. After anti-TNF treatment the upregulation of epithelial apoptotic cells in active disease restored to normal and barrier dysfunction completely recovered (25). *In vivo* histology was also able to contribute to the diagnosis and detection of IEN during surveillance colonoscopy. For CLE a meta-analysis revealed a pooled sensitivity and specificity of 91 and 97% for the differentiation of neoplastic from non-neoplastic lesions (26). Data of chromoendoscopy-guided CLE showed inconsistent results (level 4, grade C). Whereas, some studies describe a higher detection rate of IEN (3) in UC patients, other working groups did not observe a benefit over chromoendoscopy alone (27). However, the general use of this approach for surveillance cannot be recommended. Ongoing study activities with eCLE were hampered by the missing combination of the initial confocal microscope device with a newer high-definition endoscope technology due to several, unfortunately also economic reasons. Currently there is only pCLE available on the market and although there are technical and optical differences between the two systems, the usefulness of pCLE in predicting postoperative recurrence in patients with CD was shown recently (28), (level 4, grade C). Now there is a possibility to apply pCLE with nearly any commercial endoscope independent of the manufacturer. For further characterization of intestinal barrier function in IBD *in vivo* by pCLE, reliable and reproducible diagnostic criteria should be defined. The quantification of gaps, fluorescein leakage and cell shedding (5, 29) (level 3–4, grade C) are encouraging first candidates for the measurement of barrier function *in vivo* and may act as main criteria. Crypt tortuosity, distortion of crypt openings and decreased crypt density were additional observations in UC patients (30) and could potentially act as minor criteria (level 3, grade C). A number of CLE-based rating systems and scores have been published so far taking into account the degree of inflammation and the prediction of relapse (31). For the assessment of clinical outcomes or the determination of relapse rates in IBD patients under immunosuppressive therapy further research is necessary. The number of research projects investigating CLE in IBD is currently decreasing. One reason for this may be the introduction of emerging artificial intelligence systems (32) on the market, which will be part of future detection of IEN. However, for the determination of disease activity to predict relapse or therapy response in IBD there is an ongoing need for further CLE evaluation.

## Molecular Imaging

Fluorescence endoscopy (33), near-infrared fluorescence endoscopy (34) and autofluorescence endoscopy were often subsumed under molecular imaging devices. These techniques can be combined with virtual chromoendoscopy (35). However, these technologies were rather classical “red flag” technologies than real molecular imaging techniques. The years of research of the newer *in-vivo* histology techniques deliver the basis for a more detailed analysis of the underlying molecular pathways. More specific and distinct molecular imaging in advanced gastrointestinal endoscopy is the real-time visualization and binding of labeled-molecules to targeted structures on the surface of epithelial cells and the detection of this conjunction by *in vivo* histology. Probes usable for molecular imaging could be labeled antibodies, peptides, enzymes, affibodies or lectins, respectively (36). Molecular imaging is far away from widespread clinical use. However, it potentially allows a highly-individualized and specific characterization of mucosal inflammatory diseases in the future. *In vivo* studies with labeled antibodies imply a long-lasting and extensive process of approval and fulfillment of strict requirements before the use in humans is approved by regulatory authorities. The first *in vivo* application of fluorescein-labeled heptapeptides during a colonoscopy detecting colonic dysplasia was in 2008 (37). Two years later, targeting of epidermal growth factor receptor (EGFR) in colorectal cancer allowed the discrimination of neoplastic and non-neoplastic tissue areas in living animals and human tissue samples (38). One underlying signaling pathway identified a link between inflammation and tumorigenesis and was described in colitis-associated cancer (39). After demonstrating the feasibility and safety of molecular imaging in pre-malignant or malignant disease *in vivo*, further research focused on inflammatory disease with the goal to predict therapy response or relapse. The first molecular target of interest in IBD was TNF. A landmark study detecting the binding of membrane-bound TNF (mTNF) by a fluorescent-labeled adalimumab anti-TNF antibody showed that high numbers of mTNF-positive cells correlated with higher short-term response rates to treatment with the TNF-neutralizing antibody adalimumab. Patients with high numbers of mTNF-expressing cells demonstrated a higher probability of clinical response than patients with low numbers of mTNF+ cells (92 vs. 15%). The sensitivity, specificity and accuracy for the prediction of therapeutic responses were 92, 85, and 88%, respectively. Positive and negative predictive values were 85 and 92% (40). Recently, first data presented the detection of mucosal α4β7 integrin *ex vivo* with a fluorescent labeled anti-adhesion antibody vedolizumab in CD (41). In the clinical management of IBD patients, early prediction of response or failure of a planned therapy would be of utmost clinical importance. Consequently, a prompt adjustment of planned immunosuppressive therapy would be possible (42).

## Multiphoton Microscopy

Multiphoton microscopy (MPM) is one of the emerging innovative imaging technologies with the potential to visualize intestinal epithelial cells under normal and inflamed conditions without the addition of exogenous fluorescent dyes (43). The first data with MPM as a promising imaging technology in IBD revealed a clear discrimination of epithelial and immune cells and the amount of extracellular matrix (7). This label-free imaging of intestinal cellular and subcellular structures based on autofluorescence and second harmonic generation signals has therefore some advantages compared to CLE and was further developed for *in vivo* use. Recently, the first experiments in normal and inflamed murine colonic mucosa in a dextran-sulfate sodium-induced colitis model showed feasibility and a gradually deformation of the crypt architecture depending on the activity of the colitis (8). A future perspective would be the combination of MPM with a high-definition endoscope to enable the use during routine gastrointestinal endoscopy without the requirement of any exogenous labeling.

## Future Perspectives

**Figure 1:**
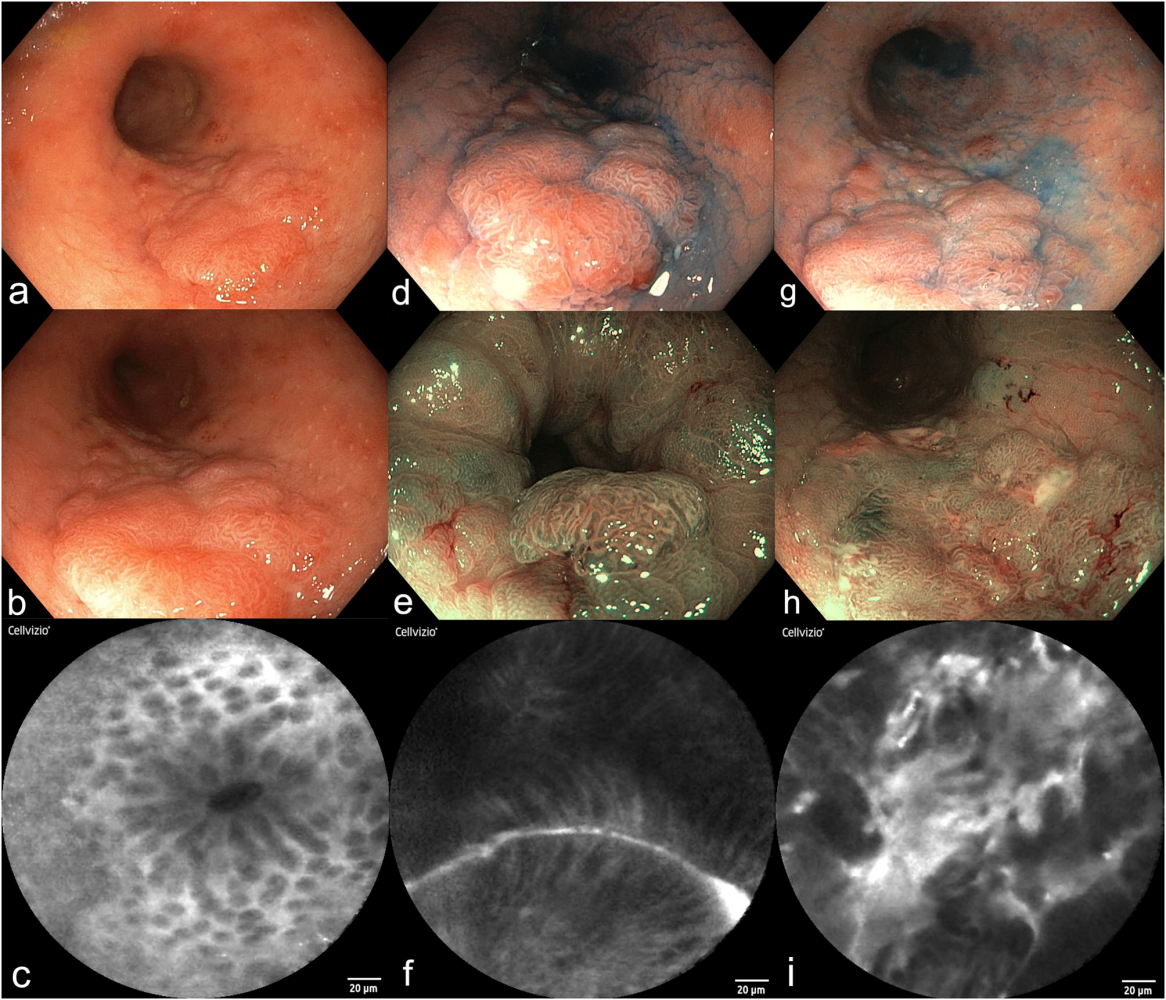
Surveillance colonoscopy in a female patient (64y) with a history of UC for 15 years **(a–i)**. **(a,b)** High-resolution video endoscopy shows a flat polypoid lesion in the sigmoid colon, size 4 × 2 cm, Paris Classification IIa+c. **(c)** Probe-based confocal laser endomicroscopy of the surrounding mucosa revealed mild inflammation and normal crypts. **(d–f)** Dye-based chromoendoscopy with indigocarmine **(d)**, narrow-band imaging [NBI, **(e)**], and pCLE **(f)** of the distal border of the polyp. A tubular structure and distorted mucosal epithelial cells become visible. **(g–i)** Dye-based chromoendoscopy with indigocarmine **(g)**, NBI **(h)**, and pCLE **(i)** of the proximal part of the polyp. **(i)** Shows high-grade intraepithelial neoplasia. Final histology of this lesion after proctocolectomy revealed well-differentiated intramucosal cancer without invasion.

On the way to an individualized endoscopic approach, a large number of technical improvements are nowadays available for patients with IBD. These include mainly high-definition endoscopy with nearly comparable efficiency compared to dye-based and virtual chromoendoscopy techniques. If upcoming clinical studies with oral intake of methylene blue prior to surveillance colonoscopy become available and confirm the additional benefit, the reservations against classical dye spraying would finally come to an end. Although CLE as the most widely used *in vivo* histology method brought extensive insight and understanding of gastrointestinal mucosal pathology, its widespread use in routine endoscopy is hampered by the lack of reimbursement and additional examination time (Figure 1). However, CLE opened the field for molecular endoscopy allowing specific targeting of surface molecules. The prediction of therapeutic response followed by prompt adjustment of targeted therapeutic strategies improve clinical decisions in complex IBD courses. MPM is an emerging new technology and the first data are now available showing *in vivo* use in an animal model. Label-free high-resolution endomicroscopy would be the logical consequence and a perfect long-term perspective for the use in patients with IBD.

## Author Contributions

All authors listed have made a substantial, direct and intellectual contribution to the work, and approved it for publication.

## Conflict of Interest

The authors declare that the research was conducted in the absence of any commercial or financial relationships that could be construed as a potential conflict of interest.
